# Preparation process of active enzymolysis polypeptides from seahorse bone meal

**DOI:** 10.1002/fsn3.125

**Published:** 2014-05-28

**Authors:** Zhanzhi Jiang, Yongjian Xu, Yuting Su

**Affiliations:** School of Marine Sciences, Ningbo University/Key Laboratory for Applied Marine Biotechnology of Ministry of EducationNingbo, 315211, China

**Keywords:** *Hippocampus trimaculatus* Leach, polypeptide, preparation process, response surface methodology

## Abstract

The preparation process of protein enzymolysis technology for the three-spot seahorse (*Hippocampus trimaculatus* Leach) degreased bone meal was developed. Two enzymes with better enzymolysis effect were selected from the five proteases, and the optimization condition of the Alkaline Protease is temperature – 54.7°C, pH – 9, duration of 6 h, the acquired rate of polypeptides was 11.77%; and that of Trypsin is temperature – 45°C, pH – 8.8, and duration of 4 h, and the rate was 11.49% by Response Surface Methodology. The strategy of compound enzymes was “Trypsin + Alkaline Protease”. The selected preparation process of active polypeptides by the compound enzymolysis technology acquired rate of polypeptides was 14.41 ± 0.16%, increased about 3% of acquired rate of polypeptides and 2.6–4.5% of the free radical scavenging rate than those of the single enzyme. The increased antioxidant capacity mainly came from the increased concentration of polypeptide in II^#^ peak, which increased about 10% of the free radical scavenging rate. The strategy of selected compound enzymes can effectively improve the utilization rate of seahorse protein.

## Introduction

The seahorse is an important resource of Chinese Tradition Medicine. Several species of the seahorses were recorded in ancient Chinese medicine books with the same pharmacological functions (Li 1988), included three-spot seahorse (*Hippocampus trimaculatus*), corona seahorse (*H. histrix*), yellow seahorse (*H. kuda*), small seahorse (*H. japonicas*) and huge seahorse (*H. Kelloggi*) (Anon 1997).

Seahorses have high content of crude protein, up to 67.9–73.56% (Yan et al. 2002; Guan et al. 2004). However, they have low-bioactive peptides (Zhang et al. 1997). One of the medicinal applications of the seahorse is wine, which cannot effectively dissolve the high content of protein components.

There are few reports about the utilization of seahorse protein. The polypeptide was the main reported product, whose functions included high-absorption speed and high-absorption rate by intestinal tract, lowering serum cholesterol, and reducing blood pressure and antioxidation (Zeng et al. 2005). Yuan et al. (2011) reported that a hydrolysis process of protein by a single enzyme and its polypeptide produce DPPH radical scavenging effect. Chen et al. (2011) extracted liquefied protein (polypeptide) from the three-spot seahorse, with the enzymatic hydrolysis rate by Alkaline protease of 67% and free radical scavenging rate of 75.59%. Both articles reported the high rates of enzymatic hydrolysis using concentration of liquefied protein in aqueous solution. However, the rate did not show the effect of enzymatic hydrolysis; only reflected the product recovery rate of seahorse protein during the process of enzymatic hydrolysis.

In this study, we tested the effects of hydrolysis degree (DH) on free amino nitrogen that would better reflect the composition of polypeptide fragments after enzyme digestion (Chen et al. 2011). In addition, we designed the experiment of compound enzymatic hydrolysis based on the optimization of the process of single enzymatic hydrolysis in order to further improve the hydrolysis yield. Furthermore, the hydrolysis polypeptide by compound enzymes would be passed through gel column chromatography system, and compared their antioxidant capacity of different separated polypeptide components.

## Materials and Methods

### Material

The adult dried three-spot seahorse (*H. trimaculatus* Leach) was purchased from a local drugstore, which was originated from artificial aquaculture by Ningbo Yonghe Aquaculture Company (Ningbo, China). The five enzymes (Papain [EC3.4.22.2], Alkaline protease [EC3.4.21.14], Trypsin [EC3.4.21.4], Pepsin [EC3.4.23.1], and Flavourzyme), DPPH (Sigma, San Francisco, CA), Pyrogallic acid, Tris, G-50 glucosan (Nanjing Jiancheng Bioengineering Co., Nanjing, Jiangsu Province, China) and other chemicals of analytical reagent were purchased from the Sinopharm Chemical Reagent Co. Ltd (Shanghai, China).

### Pretreatment

The cleaned seahorses were placed into the vacuum freezing dryer (Freezone12; Labconco Co. Ltd, Kansas City, MO) under −40°C for 48 h. Then, the freeze-dried material was crushed with a portable grinder (DFT-50A; Wenlin, Zhejiang Province, China). Due to particle size difference between the cortex and bone tissue, the bone meal passed through the 60 meshes sieve (Vibration sieve machine, AS200; Huangyan, Zhejiang Province, China) and was collected. Then, the bone meal was degreased by supercritical CO_2_ fluid extraction (SFE-CO_2_) (Speed; ASI Co. Ltd, Allentown, PA); the conditions were CO_2_ pressure of 35 MPa, extracted temperature of 58°C, time of 135 min and CO_2_-flow rate of 15 L·h^−1^ (Jiang et al. 2013).

### Crude protein in the bone meal

The content of crude protein in the degreased bone meal was determined following the semi-micro Kjeldahl method (China GB/T 5009.5-2003).

### Bone meal digestion process

A quantity of 0.30 g of the degreased bone meal was put into a 50 mL conical flask, and 10 mL distilled water was added. The water-bath temperature was raised to the predetermined levels, and the solution pH and E/S ratio were adjusted to the set values. When the process of hydrolysis reached the predetermined time, the conical flask was kept in the boiling water bath and incubated for 5 min in order to destroy the activity of enzyme and to terminate enzymatic reaction; then the aqueous solution was rapidly cooled to room temperature, centrifuged at 4030 *g* for 10 min, the supernatant solution was fixed to 30 mL. The no-bone meal control group was conducted under the same conditions and process.

### The degree of hydrolysis

The DH of the bone meal was determined by formaldehyde fixed nitrogen method (China GB/T 5009.39-2003). Ten milliliter of the hydrolysis supernatant solution was removed and placed into another conical flask and 60 mL distilled water was added. The pH value of the solution was adjusted to 8.20 and was titrated by standard 0.02 mol/L NaOH solution to pH 9.20. The number of standard NaOH consumed was recorded. The computational formula of the DH was determined from the following.


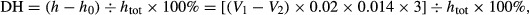


where DH: hydrolysis degree, %; *h*, *h*_0_: the content of amino nitrogen in the test group and the control group, *μ*g/mL; *h*_tot_: the content of crude protein in degreased bone meal, TN *μ*g/mL; *V*_2_, *V*_1_: standard NaOH volume consumed in the test group and the control group, respectively, mL.

### Optimization process

#### Selection of hydrolysis enzyme

Five proteases were selected for enzymolyzing seahorse bone meal. Table 1 showed their appropriate conditions of temperature, pH value, incubation time, and the enzyme–substrate ratio (E/S ratio) of 4% (Yu 2011). The DH of each enzyme was measured according to the hydrolysis process mentioned above.

**Table 1:** Different hydrolysis conditions and hydrolysis degrees of each enzyme.

Enzyme species	Temperature/°C	pH Value	Time/h	DH/%
Pepsin	37	2.0	6	4.68 ± 0.14^e^
Trypsin	50	8.5	4	10.93 ± 0.17^b^
Alkaline protease	50	9.0	4	11.52 ± 0.24^a^
Flavourzyme	50	6.5	4	8.57 ± 0.16^d^
Papain	50	7.5	4	10.13 ± 0.14^c^

a, b, c, d, e means data difference. DH, hydrolysis degree.

Two optimal enzymes (Trypsin and Alkaline protease) were selected that had a better DH than the other three. The conditions of the two enzymes were analyzed, such as temperature, pH value, incubation time and E/S ratio. Each factor and its gradients of both enzymes are as followed, temperatures of 45, 50, 55, 60°C; pH values of 7, 8, 9, 10; durations of 1, 3, 5, 7 h; E/S ratios of 1%, 2%, 3%, 4%, 5%. Each factor of every enzyme had three replicates.

#### Single enzyme process optimization

The selected two enzymes were adopted the software of Design Expert V8.0.5 (Stat-Ease, Inc., Minneapolis, MN) to determine the response surface (Central Composite Design) for optimizing the conditions of enzyme hydrolysis. The levels of experimental factors are shown in Table 2.

**Table 2:** Both enzymes and their response surface experimental design.

Level	Temperature/°C	Time/h	pH
−1.68	41.6	3.3	6.3
−1	45	4	7
0	50	5	8
1	55	6	9
−1.68	58.4	6.7	9.7

#### Compound enzyme hydrolysis

In order to obtain higher DH or smaller molecular peptides, the combined hydrolysis of both enzymes (Trypsin and Alkaline protease) was explored to form a new scheme of compound enzymes hydrolysis. Under its best condition, Akaline protease or Trypsin had hydrolyzed the seahorse bone meal at first, and then the other enzyme was added for further hydrolysis at its optimum condition.

### Polypeptide separation

After destroying the activity of enzyme, the enzymolysis solution centrifuged at 4030 *g* for 5 min, then freeze-dried polypeptide power by vacuum freezing dryer (Freezone12; Labconco Co. Ltd, USA). A quantity of 0.10 g of the dried power was put into a 10 mL PE tube, and 2 mL distilled water was added. The solution passed through the Sephadex G-50 gel column, distilled water was used for elution. The eluent was collected and the concentration of protein was detected using UV visible spectrophotometer (Puxi Corp., Beijing, China) at 280 nm wavelength. The elution curve was obtained with eluent volume as abscissa and absorbance as ordinate. The eluting peaks were collected according to the curve (eluting time).

### Determination of antioxidant polypeptide

There were two parts for determination of antioxidant polypeptide, one was to compare the antioxidant capacity among the enzymolysis polypeptide of Trypsin, Alkaline protease, and compound enzymes by DPPH method (Yuan et al. 2011); the other was to analyze the antioxidant capacity of the polypeptide of the each eluting peak from enzymolysis of the compound enzymes. The polypeptide of the eluting peaks dissolved with four concentration gradients of 0.9, 1.2, 1.5, 1.8 mg/mL for analyzing their antioxidant capacity by two methods followed.

#### Determination of DPPH free radical scavenging rate

Two milliliter of DPPH ethanol solution was put into 2 mL polypeptide solution of every gradients, placed 30 min at the room temperature. After that, absorbance (Ai) was determined with anhydrous ethanol used as reference at 517 nm wavelength. Absorbance (Ac) was determined at 517 nm of the solution with 2 mL DPPH ethanol solution and 2 mL anhydrous ethanol. Absorbance (Aj) was determined at 517 nm of the solution with 2 mL polypeptide solution and 2 mL anhydrous ethanol. The absorbance of Ai, Aj, and Ac was recorded. The computational formula of DPPH free radical scavenging rate was determined from the following (Yuan et al. 2011).


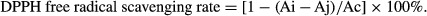


#### Determination of superoxide anion free radical scavenging rate

The method called Pyrogallic acid autoxidation method (Zhang et al. 2009). Put 4.5 mL of 50 mmol/L Tris-HCl buffer (pH 8.2), 4.2 mL distilled water and 0.3 mL of 45 mmol/L pyrogallic acid into a test tube, the absorbance (Fo) of the solution was determined at 325 nm every 30 sec. In another test tube, the 4.2 mL distilled water was placed with 1 mL polypeptide solution of every gradients and 3.2 mL distilled water, and the absorbance was recorded as F1. The computational formula of superoxide anion free radical scavenging rate was determined from the following (Zhang et al. 2009).


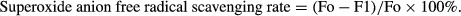


### Data analysis

With the aid of Excel software and SPSS 13.0 (Chicago, IL), the conditions and effects of hydrolysis of the seahorse bone meal and the antioxidant capacity of the enzymolysis polypeptide were analyzed and compared by the one-way analysis of variance (ANOVA).

## Results

### Hydrolysis enzyme selection

The results of using the five enzymes to hydrolyze the seahorse bone meal are shown in Table 1. The DH of the different enzymes was different; Alkaline protease was the best with the DH of 11.52%, slightly higher than those of Trypsin and Papain (*P* < 0.05), substantially higher than those of Flavourzyme and Pepsin (*P* < 0.01). The DH of Trypsin was 10.93%, slightly higher than that of Papain (*P* < 0.05). Therefore, Alkaline protease and Trypsin were selected for the next experiments.

### Single-factor experiment of single-enzyme hydrolysis

**Figure 1:**
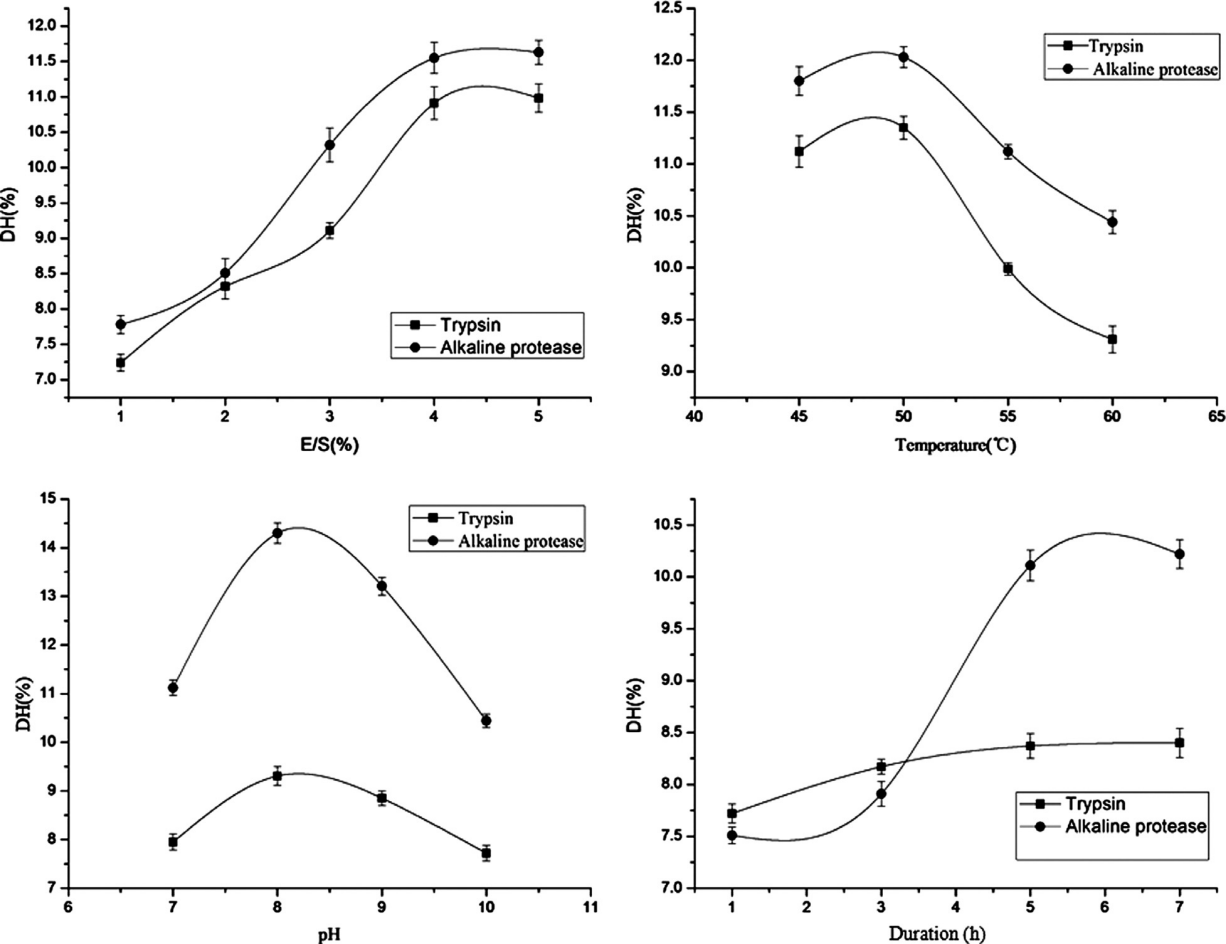
Effect of several factors on DH of two enzymes with single-factor experiment.

Through the single-factor experiment, the conditions of E/S ratio, temperature, pH value and duration of enzyme hydrolysis of Alkaline protease and Trypsin were investigated, respectively. The results are shown in Figure 1. The best enzyme/substrate (E/S) ratio was ≥4% (E/S = 4% was applied for next all experiments in this article), duration was 5–7 h, pH value was 8–9, and temperature was 50°C for both enzymes.

### Single enzyme process optimization

#### Results of response surface experiment

For optimizing the three conditions (temperature, pH value and duration) of enzyme hydrolysis, the response surface experiment design was employed. The results are shown in Table 3.

**Table 3:** The factor table and its results of the response surface experiment.

Run	Temperature/°C	Time/h	pH Value	Alkaline protease/DH%	Trypsin/DH%
1	45 (−1)	4 (−1)	7 (−1)	9.57 ± 0.09	10.22 ± 0.12
2	50 (0)	5 (0)	6.3 (−1.68)	10.24 ± 0.11	9.31 ± 0.07
3	50 (0)	5 (0)	8 (0)	13.17 ± 0.07	10.31 ± 0.09
4	41.6 (−1.68)	5 (0)	8 (0)	10.03 ± 0.13	10.67 ± 0.09
5	50 (0)	5 (0)	8 (0)	12.89 ± 0.06	10.67 ± 0.11
6	50 (0)	6.7 (1.68)	8 (0)	13.22 ± 0.12	11.60 ± 0.12
7	50 (0)	5 (0)	8 (0)	12.49 ± 0.14	11.43 ± 0.11
8	50 (0)	5 (0)	9.7 (1.68)	11.78 ± 0.15	9.99 ± 0.07
9	55 (1)	6 (1)	7 (−1)	11.13 ± 0.13	11.24 ± 0.09
10	50 (0)	5 (0)	8 (0)	12.94 ± 0.13	11.35 ± 0.12
11	45 (−1)	6 (1)	7 (−1)	11.56 ± 0.06	10.10 ± 0.09
12	50 (0)	3.3 (−1.68)	8 (0)	9.98 ± 0.11	10.44 ± 0.07
13	55 (1)	4 (−1)	7 (−1)	10.57 ± 0.09	8.74 ± 0.12
14	55 (1)	4 (−1)	9 (1)	11.98 ± 0.13	9.12 ± 0.08
15	58.4 (1.68)	5 (0)	8 (0)	11.83 ± 0.11	9.19 ± 0.14
16	55 (1)	6 (1)	9 (1)	11.08 ± 0.12	9.08 ± 0.08
17	45 (−1)	6 (1)	9 (1)	11.23 ± 0.10	10.25 ± 0.13
18	50 (0)	5 (0)	8 (0)	11.96 ± 0.07	11.24 ± 0.12
19	50 (0)	5 (0)	8 (0)	12.62 ± 0.09	11.01 ± 0.14
20	45 (−1)	4 (−1)	9 (1)	10.12 ± 0.13	11.12 ± 0.06

DH, hydrolysis degree.

Then, the regression equations of the two enzymes were obtained for Alkaline protease (a) and Trypsin (b) in the following:


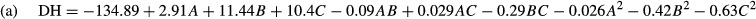



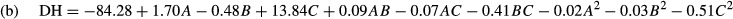


where, *A*, *B*, and *C* were temperature, duration, and pH value, respectively. *AB*, *AC*, and *BC* were the interactions.

The models’ Prob > *F*(a) (*P* value) of the two enzymes were 0.0009 and 0.0016, respectively (Table 4). Issue of Loss of models (Lack of fit) reported the probability difference between the predicted value and the actual measured value, both were not fitting, whose Prob > *F*(a) values were 0.2275 and 0.5414, respectively (Table 4). These suggested that we could use the two models for optimizing the enzyme hydrolysis process on seahorse degreased bone meal (Charin et al. 2002; Hanan et al. 2005; Zhang et al. 2009; Davoud et al. 2012; Du et al. 2012).

**Table 4:** Variance analysis of the response surface experiment.

	Squares		Freedom		Mean square		*F* value		Prob > *F*(a)	
Source of variance	Alkaline enzyme	Trypsin	Alkaline enzyme	Trypsin	Alkaline enzyme	Trypsin	Alkaline enzyme	Trypsin	Alkaline enzyme	Trypsin
Model	22.79	13.11	9	9	2.53	1.46	9.18	8.04	0.0009	0.0016
*A*-tem	2.06	2.64	1	1	2.06	2.64	7.48	14.55	0.0210	0.0034
*B*-time	4.93	0.86	1	1	4.93	0.86	17.89	4.73	0.0017	0.0547
*C*-pH	1.27	0.013	1	1	1.27	0.013	4.62	0.069	0.0572	0.7979
*AB*	1.48	1.49	1	1	1.48	1.49	5.36	8.21	0.0431	0.0168
*AC*	0.16	1.00	1	1	0.16	1.00	0.59	5.53	0.4606	0.0406
*BC*	0.68	1.35	1	1	0.68	1.35	2.48	7.47	0.1463	0.0211
*A*^2^	6.22	2.47	1	1	6.22	2.47	22.55	13.65	0.0008	0.0041
*B*^2^	2.54	0.012	1	1	2.54	0.012	9.22	0.066	0.0125	0.8019
*C*^2^	5.70	3.80	1	1	5.70	3.80	20.65	20.96	0.0011	0.0010
Lack of fit	1.85	0.86	5	5	0.37	0.17	2.03	0.91	0.2275	0.5414

The value of Prob > *F*(a) represents difference between the model and each factor; When it is >0.05, there is no effect; <0.05, there is a difference or effect, and <0.01 means a significant effect.

According to the regression equation (a), (b) and Table 4, we found that the factors *A*, *AB*, *B*^2^ had an influence (Prob > *F*(a) values <0.05), and *B*, *A*^2^, *C*^2^ had a significant influence (Prob > *F*(a) values <0.01) on the enzyme hydrolysis of Alkaline protease, but *C*, *AC*, *BC* had no significant influence (Prob > *F*(a) values >0.05); On the enzyme hydrolysis of Trypsin, the factors *AB*, *AC*, *BC* had an influence (Prob > *F*(a) values <0.05), and *A*, *A*^2^, *C*^2^ had a significant influence (Prob > *F*(a) values <0.01), but *B*, *C*, *B*^2^ has no influence (Prob > *F*(a) values >0.05). Regression equation obtained after optimization:


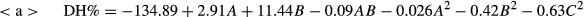



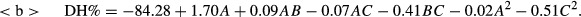


For Alkaline enzyme, the factors of temperature and duration had large effect on its hydrolysis to the seahorse bone meal, followed by pH value; however, for Trypsin, the temperature factor was the most important, followed by pH value and then duration.

#### Analysis and optimization of interaction

**Figure 2:**
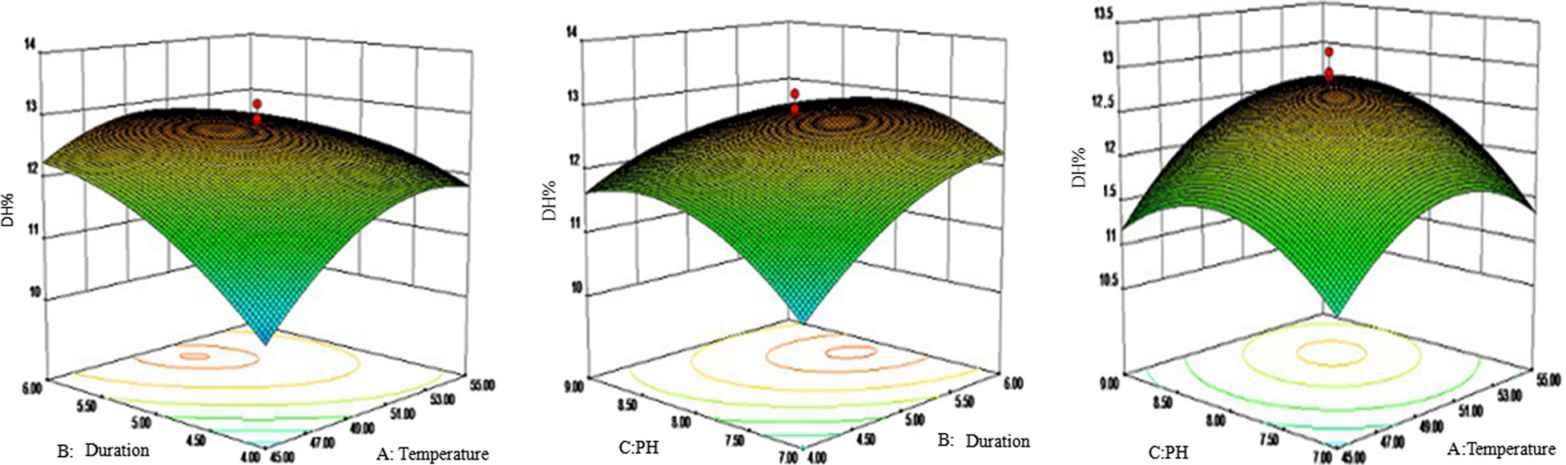
The interactions among duration, temperature, and pH value in response surface experiment of Alkaline protease.

**Figure 3:**
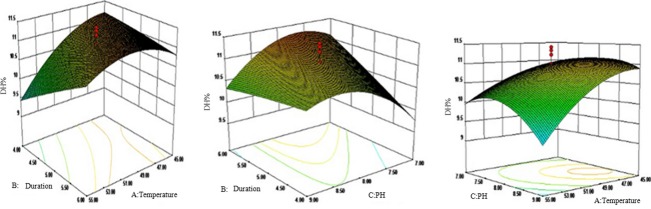
The interactions among duration, temperature, and pH value in response surface experiment of Trypsin.

According to the analysis results (Figs. 2, 3) of the response surface experiment, we could solve the optimization equations <a> and <b> with Design-Expert software used the DH as the evaluation index, and attained the optimum combination factors were, Alkaline protease, temperature 54.7°C, duration of 6 h and pH value 8.99, the predicted DH to seahorse bone meal was 11.77%; Trypsin, 45°C, 4 h and 8.78, the predicted DH was 11.49%.

#### Compound enzyme hydrolysis

In order to facilitate the operation, we corrected the pH values of enzyme hydrolysis as 9.0 for Alkaline protease and 8.8 for Trypsin (Charin et al. 2002; Hanan et al. 2005; Srimanta et al. 2011; Yin et al. 2011). The compound enzyme combinations and their hydrolysis results are shown in Table 5. Through one-way ANOVA method, four treatments and their hydrolysis results were analyzed and compared. In the treatments of single enzyme (Experiment 3 and 4, Table 5), there were no differences in DH% between the predicted values and the measured values. In the treatments about compound enzyme combinations, the DHs were 2–3% higher than those in single-enzyme treatments (*P* < 0.01); and the best combination strategy was Trypsin + Alkaline protease, that is, Trypsin was employed firstly, Alkaline protease was the second step, cohydrolyzed the seahorse bone meal (Experiment 3 and 4, Table 5).

**Table 5:** Compound enzyme combinations and their hydrolysis results.

No.	Enzyme combinations	Temperature/°C	Time/h	pH	E/S	DH%
1	Alkaline protease + Trypsin	54.7–45	6–4	9–8.8	4%	13.73 ± 0.16^a^
2	Trypsin + Alkaline protease	45–54.7	4–6	8.8–9		14.41 ± 0.16^b^
3	Alkaline protease	54.7	6	9		11.82 ± 0.14^c^
4	Trypsin	45	4	8.8		11.45 ± 0.11^c^

a, b, c means data difference. DH, hydrolysis degree.

### Polypeptide separation by gel column

**Figure 4:**
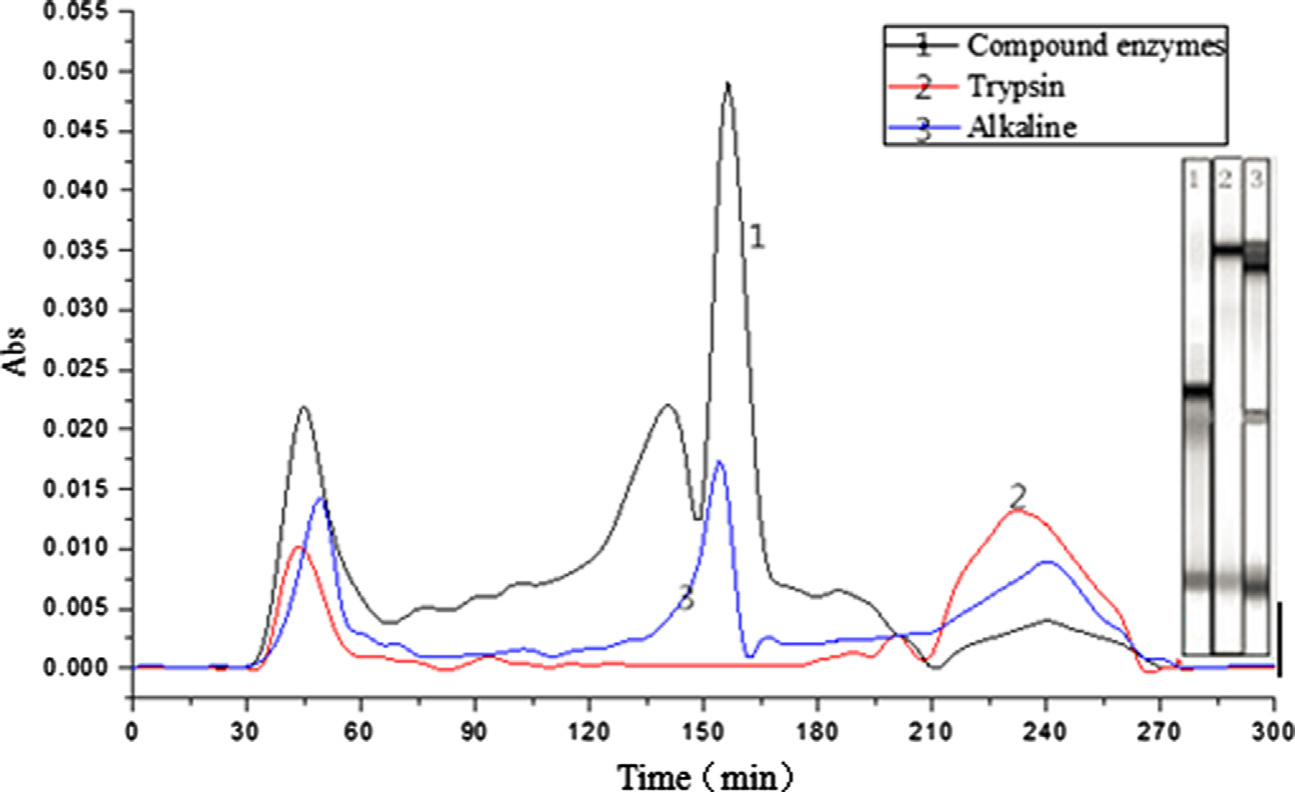
The enzymolysis polypeptide compositions of Trypsin, Alkaline protease, and compound enzymes by gel column

The polypeptide compositions of the enzymolysis solutions by Trypsin, Alkaline protease, and compound enzymes are shown in Figure 4. Seen from the figure, there were several obvious peaks in each curve. According to the eluting time (Table 6) (deleting the issues of Relative concentration <0.001 and Peak area <1000), the polypeptide of three peaks in the curve of compound enzymes was collected separately, and named I^#^ peak, II^#^ peak, and III^#^ peak for 30–68 min, 68–210 min, and 210–270 min, respectively.

**Table 6:** Characteristics of the three eluting curves in gel column.

Curve 1			Curve 2			Curve 3		
Eluting time (min)	Relative concentration	Peak area	Eluting time (min)	Relative concentration	Peak area	Eluting time (min)	Relative concentration	Peak area
30.476	0.001253	11500	33.776	0.001053	1150	27.879	0.001251	1476
42.743	8.712	110,426,830	44.766	8.977	111,268,30	50.174	10.458	136,742,00
47.343	13.85	1165,814,02	58.443	0.0461	554,39	75.214	0.0246	258,93
68.871	0.04381	1524,35	96.445	0.064	589,31	110.436	0.00147	142,93
76.412	0.2164	1258,931	190.663	0.06401	645,77	153.687	11.976	157,451,24
80.463	0.05401	1646,39	195.312	0.04334	522,50	162.142	0.00163	144,86
140.011	28.53	1341,506,21	202.699	0.1446	944,87	165.223	0.0217	186,69
149.124	9.476	1124,574,51	208.946	0.0207	8766	240.866	7.677	974,582,0
158.769	37.66	1450,719,49	231.969	9.896	128,755,60	275.489	0.0012	1001
196.669	0.9574	1114,586,2	267.449	0.00103	976			
210.568	0.001108	11029						
241.769	5.98	1715,647,9						
270.149	0.001176	11077						

### Antioxidant capacity of enzymolysis polypeptide

#### Three enzymolysis polypeptides

**Figure 5:**
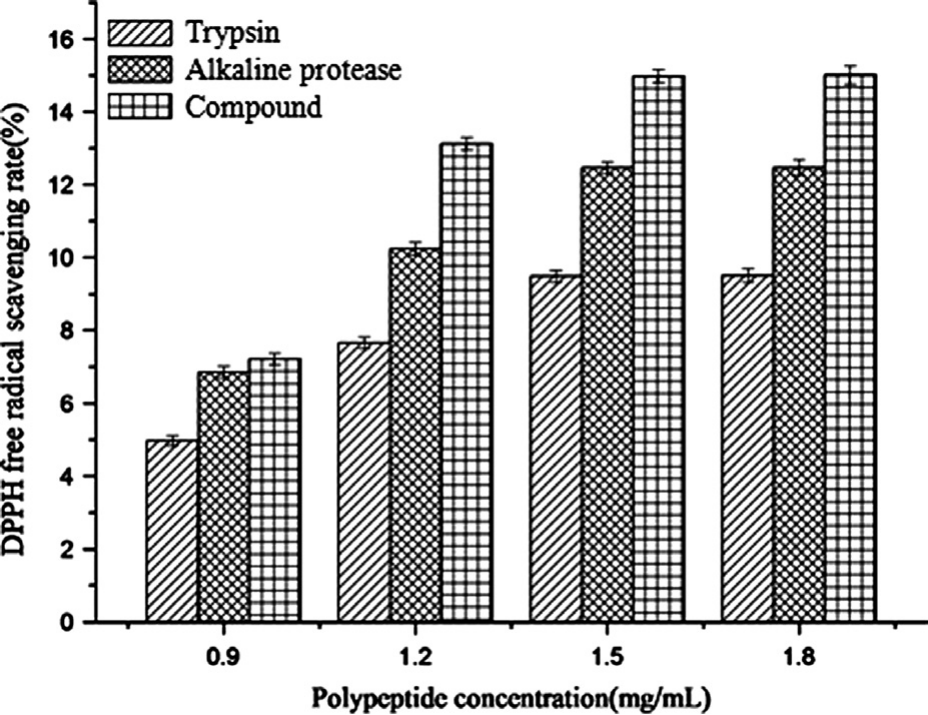
The DPPH free radical scavenging rate of three enzymolysis polypeptides by Trypsin, Alkaline protease, and compound enzymes

The DPPH free radical scavenging rate of three enzymolysis polypeptides by Trypsin, Alkaline protease, and compound enzymes are shown in Figure 5. In 0.9 mg/mL polypeptide, the scavenging rate was low, and no difference between Alkaline protease and compound enzymes were observed. With the increase in the concentration, the rates increased. When added up to 1.5 mg/mL, the rate was maximum, and they were obviously different among three enzymes (*P* < 0.05); the average scavenging rate of enzymolysis polypeptide by compound enzymes was 2.6% and 4.5% higher than that of Trypsin and Alkaline protease, respectively.

#### Three polypeptides peaks of compound enzymes

##### DPPH free radical scavenging rate

**Figure 6:**
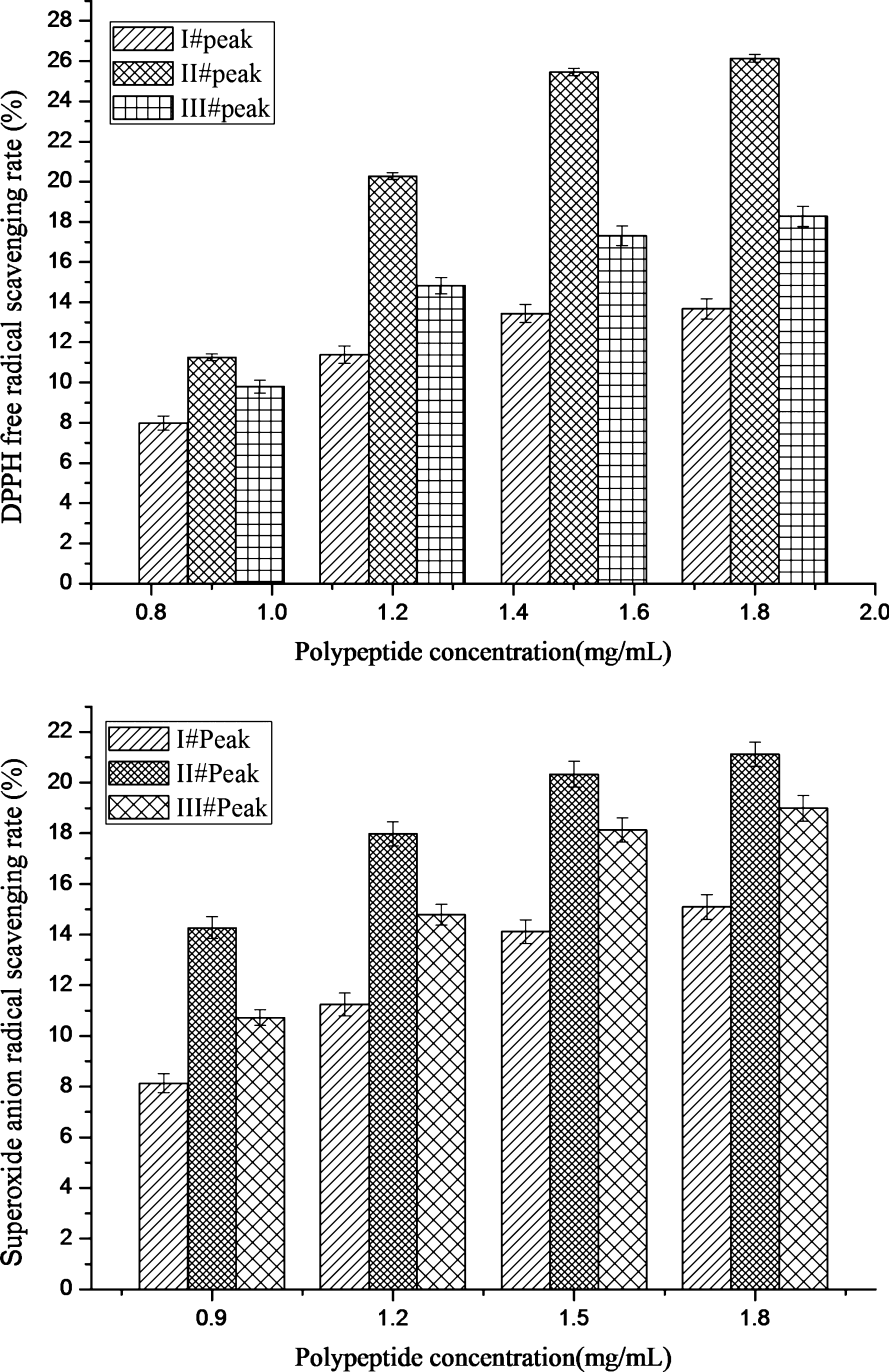
DPPH and superoxide anion free radical scavenging rate of enzymolysis polypeptide of three peaks in the compound enzymes curve

Figure 6 showed comparison of DPPH free radical scavenging rate with the polypeptide of three peaks in the curve 1. The trend was similar to Figure 5. When the concentration was 1.5 mg/mL, the maximum rate was 13.67 ± 0.32%, 26.14 ± 0.47%, and 18.28 ± 0.51% for I^#^, II^#^, and III^#^ peak, respectively. Comparing with Figure 5, only the II^#^ peak was much higher than its equivalence in free radical scavenging, and increased about 10% in the 1.5 mg/mL concentration.

##### Superoxide anion free radical scavenging rate

The comparison of superoxide anion free radical scavenging rate was shown in Figure 6. The trend was similar to DPPH. When the concentration was 1.5 mg/mL, the maximum rate was 15.09 ± 0.33%, 21.13 ± 0.42%, and 18.99 ± 0.51% for I^#^, II^#^, and III^#^ peak, respectively.

## Discussion and Conclusions

In this study, we evaluated the DH and found that the DH not only reflected the quality of enzymolysis but also the average molecular weight of polypeptides product, as the average length of enzymolysis polypeptides chain was 1/DH% (Yu 2011). DH analysis was relatively simple and easy to conduct, and does not need complex reagents and expensive instruments (Jiang et al. 2013).

Using Response Surface Methodology to optimize the hydrolysis process allows us to more accurately assess the conditions of two enzymes, and further affect the compound enzymatic process (Table 5). At present, only few reports involved the selection and optimization of enzyme hydrolysis process for seahorse production. Yuan et al. (2011) and Chen et al. (2011) reported the hydrolysis process and its antioxidant capacity of *H. trimaculatus*, but they did not involve several enzyme combinations. The compound enzymatic process could produce more quantity and smaller molecular weight polypeptide (Yu 2011). Yu (2011) studied the effect of enzymatic hydrolysis on Hairy Antler by Alkaline protease and Trypsin, through orthogonal test. The DH was 18.03% by single-enzyme hydrolysis and 21.41% by “Trypsin + Alkaline protease” compound enzymatic hydrolysis. Similar results attained in this experiment, the combination of Trypsin and Alkaline protease cohydrolyzed the seahorse bone meal, the DH was 14.41% more 2–3% than that of single enzyme. Because the hydrolysis proteases in this experiment are all endoproteinase, which are exclusive and specific to peptide bond, meaning that the peptide fragments produced by different enzymes are different and fixed. Several enzymes combination has the comprehensive advantages to protein hydrolysis and produce more polypeptide (Aaslyng et al. 1999a,b; Chowdary et al. 2000).

In this article, another technology employed was related to increase DH. CO_2_ supercritical fluid extraction (CO_2_-SFE) technology was employed to degrease seahorse bone meal, which was the material for enzyme hydrolysis process of polypeptides. Despite no reports involved in the effect of CO_2_-SFE on the DH of polypeptide, several reports detected 17 free fatty acids in the enzymatic hydrolysate, which would cause pH value to decrease in the system (Yang et al. 2008). However, both Trypsin and Alkaline protease have better functions in the alkaline environment, the pH value decreased would not be conducive to control the stability of enzymatic hydrolysis conditions, thus affecting the enzymatic hydrolysis yield.

Not only the compound enzymolysis increased the hydrolysis yield but it also could produce more number of smaller molecular peptides, which would derive from that the eluting time was shorter (Fig. 4 and Table 6), and increased the antioxidant capacity of the enzymolysis polypeptide. The increased antioxidant capacity mainly came from the increased concentration of polypeptide in II^#^ peak. Composition and structure of the polypeptide in II^#^ peak need more research.
